# Cardiometabolic Risk and Biomarker Trajectories Among Older Adults: Findings From the Health and Retirement Study

**DOI:** 10.1093/geroni/igaa057.1386

**Published:** 2020-12-16

**Authors:** Qiao Wu, Eileen Crimmins, Jennifer Ailshire, Jung Ki Kim, Erfei Zhao

**Affiliations:** University of Southern California, Los Angeles, California, United States

## Abstract

The deterioration of the cardiovascular system is a process associated with aging. Most of the prior works have examined changes in cardiometabolic risk (CMR) while aging at the population level using cross-sectional data, but we study within-person changes for total CMR and separate risk factors, including pulse pressure, resting heart rate, C-reactive protein, glycosylated hemoglobin (HbA1c), high-density lipoprotein cholesterol, total cholesterol, waist circumference, and obesity. We examine 8-year changes (from 2006 to 2014) among respondents from the Health and Retirement Study biomarker sample (n=19,776). We use growth curve models to identify differences at baseline and the changes while aging, by age, gender, race/ethnicity, and education. Blacks, the old-old, the less educated, and current smokers have higher baseline CMR. The total CMR increases while people age over 8 years. HbA1c, waist circumference, and pulse pressure increase significantly with age. A reduction in total cholesterol can be observed and is likely due to medication. The CMR increase is no longer significant after accounting for socioeconomic status. The next step of this study is to focus on the disparity of risk distribution, in order to identify the individuals that are most in need of specific care and support.

